# A comparative whole genome analysis of *Helicobacter pylori* from a human dense South Asian setting

**DOI:** 10.1111/hel.12766

**Published:** 2020-10-18

**Authors:** Shamsul Qumar, Trang Hoa Nguyen, Shamsun Nahar, Nishat Sarker, Stephen Baker, Dieter Bulach, Niyaz Ahmed, Motiur Rahman

**Affiliations:** ^1^ Department of Biotechnology and Bioinformatics University of Hyderabad Hyderabad India; ^2^ The Hospital for Tropical Diseases Oxford University Clinical Research Unit Ho Chi Minh City Vietnam; ^3^ International Center for Diarrhoeal Disease Research Bangladesh Dhaka Bangladesh; ^4^ Centre for Tropical Medicine and Global Health Nuffield Department of Clinical Medicine Oxford University Oxford UK; ^5^ Cambridge Institute of Therapeutic Immunology & Infectious Disease Cambridge University Cambridge UK; ^6^ The University of Melbourne and The Peter Doherty Institute for Infection and Immunity Melbourne VIC Australia

**Keywords:** genome sequencing, *H pylori*, outer membrane proteins, phylogeny, virulence

## Abstract

*Helicobacter pylori*, a Gram‐negative bacterium, is associated with a wide range of gastric diseases such as gastritis, duodenal ulcer, and gastric cancer. The prevalence of *H pylori* and risk of disease vary in different parts of the world based on the prevailing bacterial lineage. Here, we present a contextual and comparative genomics analysis of 20 clinical isolates of *H pylori* from patients in Bangladesh. Despite a uniform host ethnicity (Bengali), isolates were classified as being part of the HpAsia2 (50%) or HpEurope (50%) population. Out of twenty isolates, eighteen isolates were *cagA* positive, with two HpEurope isolates being *cagA* negative, three EPIYA motif patterns (AB, AB‐C, and ABC‐C) were observed among the *cagA*‐positive isolates. Three *vacA* genotypes were observed with the s1m1i1dic1 genotype observed in 75% of isolates; the s1m2i1d1c2 and s2m2i2d2c2 genotypes were found to be 15% and 10% of isolates, respectively. The non‐virulent genotypes s2m2i2d2c2 was only observed in HpEurope population isolates. Genotypic analysis of *oipA* gene, present in all isolates, revealed five different patterns of the CT repeat; all HpAsia2 isolates were in “ON” while 20% of HpEurope isolates were genotypically “OFF.” The three blood group antigen binding adhesins encoded genes (*bab* genes) examined and we observed that the most common genotype was (*babA*/*babB*/‐) found in eight isolates, notably six were HpAsia2 isolates. The *babA* gene was found in all HpAsia2 isolates but present in only half of the HpEurope isolates. In silico antibiotic susceptibility analysis revealed that 40% of the strains were multi‐drug resistant. Mutations associated with resistance to metronidazole, fluoroquinolone, and clarithromycin were detected 90%, 45%, and 5%, respectively, in *H pylori* strain. In conclusion, it is evident that two populations of *H pylori* with similar antibiotic profiles are predominant in Bangladesh, and it appears that genotypically the HpAisa2 isolates are potentially more virulent than the HpEurope isolates.

## 1 INTRODUCTION

*Helicobacter pylori* is a highly successful human pathogen that colonizes the human gastric mucosa of over half of the world's population. The prevalence of *H pylori* infection appears to be higher in developing countries than developed countries and its prevalence vary between populations and between groups within the same population.^1^, ^2^ The prevalence in Asia, Africa, varies from 54.7% to 79.1%, in North and South America the prevalence is 37.1% and 63.4% and in Europe, the prevalence is averages 47.0%.^3^ Bangladesh, one of the low socioeconomic country of South Asia with more than 160 million inhabitants, is the eight most populous country in the world. In a study, a high prevalence of *H pylori* (92%) was reported in Bangladeshi population.^4^, ^5^ Furthermore, *H pylori* infection in Bangladesh was reported to be significantly higher in smokers as compared to non‐smokers or subjects below 15 years of age.^6^ Despite high occurrence rate of *H pylori* infection in Bangladesh, the incidence of gastric cancer is low as in other developing countries.^7^

*Helicobacter pylori* has been co‐evolving with humans for more than 60 000 years.^8^ This long intimate symbiotic association of *H pylori* with human has been led to the emergence of different genotypes by accumulation of host specific adaptive changes over the period^9^ and is characterized by distinct genotypes that predominate in different geographical regions around the world.^10^ Moreover, the occurrence frequency and severity of gastric illnesses associated with *H pylori* are found to be strongly associated with the dominant genotype in that region. For example, the incidences of gastric cancer is higher in East Asian countries such as Japan and Korea when compared to Western countries.^11^

*Helicobacter pylori* can colonize a host during infancy establishing a chronic infection that can persist for decades, if not eradicated.^12^ The bacterium has numerous mechanisms to manipulate and evade host defenses to ensure its stomach survival. To counter the acidic pH in the stomach, *H pylori* utilizes urease enzyme that forms a cloud of neutral micro‐environment around the bacterium.^13^ CagPAI (cytotoxin‐associated gene pathogenicity island) containing several virulence genes that trigger abnormal cellular signals is considered to be the most important risk factor for *H pylori*‐associated gastric cancer. CagA, the most important virulence factor of CagPAI, plays a crucial role in *H pylori* pathogenesis. Comparative analysis revealed a significant functional difference in East Asian *cagA* which showed to induce higher pro‐inflammatory secretions as compared to Western *cagA*. Furthermore, *cagA* activates a number of signal transduction pathways that bind and disrupt the function of epithelial junctions, leading to aberrations in functioning of tight junction, cell polarity, and cell differentiation.^14^ *H pylori* also produces vacuolating cytotoxin A (*vacA*), which after entering in host cells by endocytosis, induces various cellular activities, including membrane channel formation, cytochorome c release leading to cell death, and cell membrane receptor binding, which initiates a proinflammotory response.^15^ Similar differences in its functionality like *cagA* have also been associated with *vacA* which is believed to be the result of host specific adaptive changes.^16^ The gene encoding *vacA* shows allelic diversity in its signal (s) regions (alleles: s1 and s2) and middle regions (alleles: m1 and m2). In vitro experiments showed s1m1 strains induce cell vacuolation more frequently than s1m2 and s2m2, from which it was inferred that the s1m1 was more cytotoxic.^17^ Allelic differences in the intermediate (i) region (alleles: i1 and i2) are suggested to be correlated with severity of disease as compared to the “s” and “m” regions.^18^ The more frequently observed deletions in the VacA have been classified and it has been observed that the deletion of 69‐81 bp (d2) type is less virulent than the no deletion (d1) type.^19^

The outer membrane proteins of *H pylori* are considered to be possible virulence factors. OipA (outer inflammatory protein), one member of this large protein family, is involved in bacterial adherence to the gastric epithelial cells and in mucosal inflammation.^20^ Additionally OipA is associated with interleukin (IL)‐8 induction, mucosal damage and with duodenal ulcer.^20^ In addition to this, the *bab* genes have been shown to be positively correlated with gastric cancer and duodenal ulcers.^21^, ^22^ Similar to other *H pylori* OMPs, BabA has two closely related paralogues, BabB (also known as HopT) and BabC (also known as HopU), although these paralogues are not well characterized.

Inappropriate and irrational use of antibiotics against infectious diseases has already resulted in the emergence of multi‐drug‐resistant bacteria globally. Currently, triple therapy comprising two antibiotics and a proton pump inhibitor is used as eradication regime for *H pylori*. However, increasing antibiotic resistance trend to metronidazole, clarithromycin, and fluoroquinolones in *H pylori* has decreased the success rate of its eradication. Inactivation mutations such as frameshift and nonsense mutations, insertions, and deletions of the *rdxA* and *frxA* genes suggested to confer resistance to metronidazole in *H pylori*.^23^ In addition, amino acid substitutions in *rdxA* are also suggested to confer resistance to metronidazole in *H pylori*.^24^, ^25^, ^26^, ^27^ On the other hand, the mechanism of fluoroquinolone resistance in *H pylori* has been associated to the mutations in quinolone resistance determining regions (QRDR) of the *gyrA*.^28^ Moreover, the strategy employed by *H pylori* for clarithromycin resistance has been elucidated mainly due to mutations at nucleotide positions (A2142G, A2143G, A2142C, A2146, A2147G, and G2224A) in the *23S rRNA*.^29^, ^30^, ^31^

Despite high prevalence of *H pylori* in Bangladesh, little is known about the local lineages or the local prevalence of particular genotypes for *cagA* or *vacA* and *babA* genotypes and any associations with disease severity.^32^ Therefore, this study was directed to evaluate the prevalence of distinct genotypes of *cagA*, *vacA*, and *babA/B* and other virulence factors together with its genotype‐based antibiotic resistance profiles in prevailing Bangladeshi *H pylori* lineages and also to find the association with clinical outcome using a genomics‐based approach to characterize genotypes and infer lineage classification. The findings would also provide a better understanding of the prevalence of antibiotic‐resistant *H pylori* strains and its genotypic molecular mechanisms to facilitate the designing of more rational and effective combinatorial antibiotics therapy for eradication of *H pylori* infection. These data enable contextualization and comparison of drug‐resistant *H pylori*‐associated disease in Bangladesh with disease in other parts of the world.

## 2 MATERIALS AND METHODS

The genome sequences for each of 20 randomly selected *H pylori* isolates collected from a cohort of 174 (125 adult and 49 children) *H pylori*‐positive symptomatic or asymptomatic patients were determined (File S1). Out of 2010 *H pylori* isolates, 1% were selected in a two‐step randomization process. In the first step, 20 patients (out of 174) were selected randomly and in the second step one isolate from each patient (from ten single colony isolates stored for each patient) was selected. The selected isolates were sub‐cultured in selective medium (BHI‐7.5% sheep blood plate, 0.4% isovitalex, 0.4% DENT supplement) under microaerophilic conditions (5% O_2_; 15% CO_2_; 80% N_2_) at 37°C for 3‐5 days.^32^ Identity of the isolates was confirmed by mass spectrometry using MALDI TOF (Bruker, Germany). Genomic DNA was prepared from confluent growth using a commercial DNA extraction kit (Qiagen DNA Mini kit, Germany). Genomic library was prepared using Nextera DNA sample preparation kit (Illumina, San Diego, CA, USA) and sequenced on an Illumina MiSeq platform at the Oxford University Clinical Research Unit, Ho Chi Minh City, Vietnam, using a V3 600 cycles, paired‐end kit (Illumina).

### 2.1 Bacterial genome assembly and annotation

Paired Fastq files obtained for each isolate were processed as follows. Low‐quality bases were removed and trimmed using the NGS QC toolkit^33^ and FAST‐X Toolkit (http://hannonlab.cshl.edu/fastx_toolkit), respectively. Read sets were then assembled using SPAdes to produce a draft genome sequence for each isolate.^34^ Contigs were reordered to be consistent with the genome of *H pylori* 26695 using Contig Layout Authenticator (CLA).^35^ Gene prediction and annotation of the assembled draft genomes were carried out by using Prokka.^36^ The Artemis genome viewer was used to access specific annotated features in each of the draft genome sequences.^37^ tRNA and rRNA were identified in the draft genomes using tRNAScan and RNAmmer, respectively.^38^, ^39^ The identification of phage‐related regions was carried out using PHASTER.^40^ In addition, sequences of CagPAI‐positive and CagPAI‐negative isolates were aligned against *H pylori* 26695 (Typical HpEurope) and *H pylori* F57 (Typical HpEastAsia) reference strains using Blast Ring Image Generator (BRIG).^41^

### 2.2 Phylogenetic analysis

Thirty‐one^31^ reference *H pylori* genome sequences representing different population/lineages were downloaded from the National Center for Biotechnology Information (NCBI) (Listed in Table S1) database. All sequences from the present study and the reference sequences were used to construct whole genome‐based phylogenetic tree using Harvest.^42^ The *H pylori* 26695 genome sequence was used as a reference for the core genome alignment. The tool was run to build a core genome‐based phylogenetic tree that excluded recombination regions as previously done by Kumar et al^43^ The phylogenetic tree was visualized in interactive tree of life (iTOL).^44^

### 2.3 Core genome and pan genome analysis

OrthoMCL was used to identify orthologous gene clusters using predicted protein sequences of all studied isolates (minimum threshold of 50 amino acid in length with identity and e‐value parameters were at 70% and 0.00001, respectively).^45^ The identified genes were aligned against the EggNOG database to define their functional categories. Genes contained more than one domain of distinct categories were classified as multiple class genes. The functional categories were graphically represented using R (http://www.R-project.org). Genes without appropriate hit against the database were classified as unknown genes. Furthermore, *H pylori* isolate‐specific genes were also identified using OrthMCL followed by an in‐house perl script. Similarly, the functionally categorized strain‐specific genes by EggNOG were depicted graphically using R. In addition, a floral Venn diagram depicted the number of core genes and isolate‐specific genes was produced using R. In addition to this, the core and specific gene content of HpAsia2 and HpEurope strains were also analyzed separately using OrthoMCL with the above‐mentioned parameters.

### 2.4 Identification of virulence‐related and outer membrane proteins

The amino acid sequences of the predicted genes were compared with the *H pylori* virulence genes listed in VFDB.^46^ Similarly, major OMPs from *H pylori* J99 were used to detect the genes encoding these protein in each of the isolates as described previously^47^ using BLASTp. Identity and coverage 80% and 70%, respectively, was used as the detection threshold.^48^ Among the virulence genes, we mainly focused on the presence and absence of *BabA*, *BabB*, and *BabC* gene and translation potential of a major OMP, *OipA* in the isolates.

### 2.5 Phylogenetic analysis of *CagA* and *VacA* genotypes

The nucleotide sequences of the *cagA* and *vacA* genes from the genomes of the isolates and from the genomes of ten representative *H pylori* genomes from a range of genotypes from NCBI (Table S1) were extracted and aligned using CLUSTALW as implemented in MEGA 5.2.^49^ These alignments were then utilized to construct *cagA* and *vacA* gene‐based phylogenetic tree using neighbor‐joining algorithm with 1000 bootstraps values. The output tree was visualized in iTOL in each instance. The multiple sequence alignments were also used for the allelic/genotypic classification of the *cagA* and *vacA* gene present in each isolate.

### 2.6 In silico analysis of antibiotic susceptibility

Blastn search was performed to extract *rdxA*, *frxA*, *gyrA*, and *23S rRNA* nucleotide sequences from each strain using reference genes *hp0954*, *hp0642*, *hp0701*, and *hpr01*, respectively, of *H pylori* 26695. Extracted *rdxA*, *frxA*, and *gyrA* gene sequences were then translated into amino acid sequences prior to alignment. Sequence alignment of extracted *23S rRNA* gene of each strain was performed at nucleotide level. Aligned sequences were then compared with reference sequence of *H pylori* 26695 to examine for reported and novel mutations.

## 3 RESULTS

### 3.1 Patient information and strains isolation

Twenty *H pylori* isolates, one each from 20 patients were selected through 2‐step randomization procedure and were analyzed. The average age of the patients was 29 years; 7‐59 years range. Seventy percent (14/20) of the patients were male. None of the patients had any other concomitant illness. Socioeconomically, 15% (3/20), 25% (5/20), and 60% (12/20) of the patients were from a high (>5000 taka/month), medium (2500‐5000 taka/month), or low income group (<2500 taka/month), respectively. More than half, 65% (13/20), of the patients had basic literacy skills (5‐10 years schooling) and 35% were illiterate. Twenty‐five‐percent (5/20) of the patients were smokers and none reported raw tobacco chewing or alcohol intake. Thirty percent (6/20) of the patients reported dyspepsia, heartburn, and occasional H2 blocker use. Among the isolates 12 (60%) were from gastric antrum, seven (35%) from gastric body and one (5%) were from gastric juice.

### 3.2 Genome characteristics

Table 1 presents the general characteristics for each of the 20 genome sequences. There were 28‐70 contigs with a high (40×–1070×) genome coverage. The mean genome size was 1.6 Mb and the mean G + C content was 38.9%. Each genome contains between 1586 and 1710 annotated CDS with an average ~92% of the genome is used for the coding region.

**Table 1:** Genome statistics of the whole genome sequences of the 20 *H pylori* isolates in this study

Strains designation	Genome coverage	No. of contigs	Genome size	No. of CDS	Coding percentage	G + C percentage	rRNA	tRNA	EPIYA Motif
17A6	92	61	1643694	1710	91.9	38.85	3	36	AB‐C
19B6	84	38	1600763	1601	92.2	39.01	4	37	AB‐C
25b2	140	41	1641783	1695	92.5	38.79	4	36	AB‐C
20A8	77	40	1659973	1692	92.4	38.9	3	37	AB‐C
28B4	82	22	1616466	1635	92.5	39.02	4	37	AB‐C
37A5	111	70	1662458	1700	91.7	38.85	3	36	AB‐C
40A6	139	53	1640998	1660	92.1	38.84	3	37	AB‐C
43a2	40	39	1635561	1648	92.2	38.86	3	37	AB‐C
44A4	92	35	1591152	1608	92.5	39.04	4	36	AB‐C
59a9	80	42	1641723	1694	92.6	38.8	4	36	AB‐C
60A7	61	46	1642721	1654	92.1	38.85	4	37	AB
61A5	78	36	1602458	1600	91.3	38.92	6	36	AB‐C
149A3	1070	49	1634281	1672	92.5	38.9	3	36	AB‐CC
86A5	103	28	1616047	1640	92.3	38.92	4	37	AB‐C
88A4	107	43	1617524	1651	92.3	38.9	4	37	AB‐C
89B9	80	33	1619489	1614	92.2	38.91	4	37	AB‐C
GJ906	79	30	1612378	1639	92.3	38.9	4	37	AB‐C
S106A3	51	40	1609865	1624	92.4	39.03	4	37	AB‐C
S108A3	68	41	1645721	1668	91.9	38.84	5	36	AB‐CC
152B5	89	28	1579779	1586	92.3	38.99	4	36	AB‐C

Fifty‐five percent (11/20) of the isolate genomes harbored one/two either intact, incomplete phage sequence (4.8‐52.6 kb). The phage sequences consisting of between 9 and 39 CDSs, encode helicase, terminase, integrase, transposase, methylase, and other hypothetical proteins in addition to phage‐related genes (Table S2).

The OrthoMCL analysis identified 30 267 proteins forming 1765 orthologus gene clusters. The core genome consisted of 1256 orthologous gene clusters. Out of 1256 core gene clusters, 1094 clusters could be assigned to different functional classes by EggNOG database (Figure S1A). A high proportion (~11%, 117/1094) of genes belong to J (translation, ribosomal structure and biogenesis) and M (cell membrane/envelope biogenesis) functional classes. Approximately 15% (168/1094) of genes were classified as unknown where the function could not be predicted. In addition, 212 unique genes were identified in the 20 isolates and only 99 of these were assigned to various classes by EggNOG database. The majority were classified the S (hypothetical) or L (replication, recombination, and repair) functional classes (Figure S1B). A small but variable number of unique genes were identified for each isolate (Figure S1C). Moreover, an additional performance of OrthoMCL analysis predicted 1712 and 1669 orthologous gene clusters in HpAsia2 and HpEurope strains, respectively. The core of HpAsia2 contained 1308 genes while 1295 genes were present in the core of HpEurope. In addition to this, 142 and 210 genes were identified as unique in HpAsia2 and HpEurope strains, respectively. In both lineages, majority of the unique genes were classified to L (replication, recombination and repair) and S (function unknown) classes.

### 3.3 Core genome phylogenetic analysis

**Figure 1:**
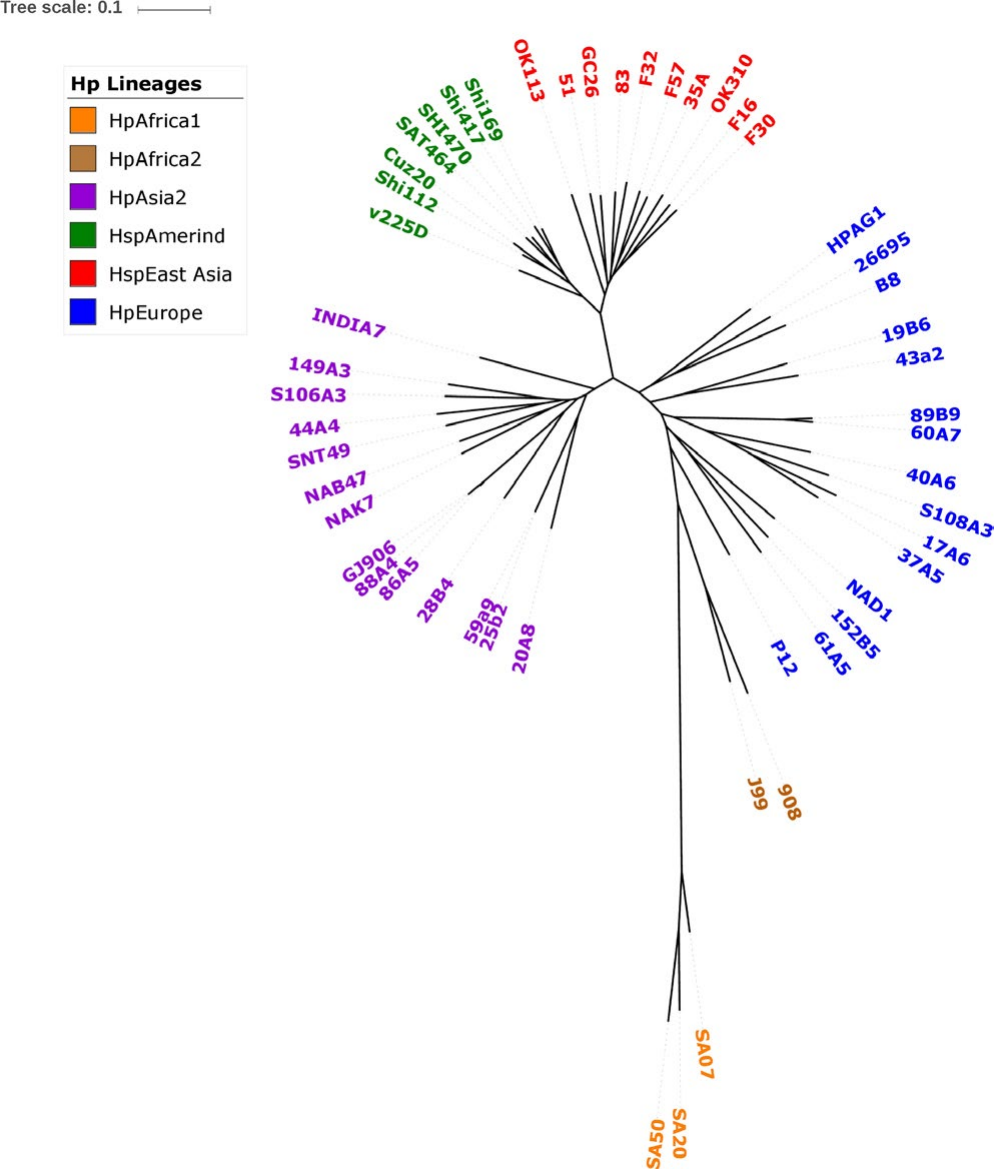
Whole genome core SNP‐based phylogenetic tree of the 20 Bangladeshi *H pylori* isolates and 31 *H pylori* reference genomes. The analysis was done using Harvest tool and the output of Harvest was visualized using iTOL

We used whole genome SNP‐based phylogenetic analysis to infer the lineage of each of the isolates. All isolates from the present study were separated into two distinct population; 50% (10/20) of the isolates belonged to HpAsia2 (50%) and 50% (10/20) to HpEurope cluster despite being from one ethnicity (Figure 1).

### 3.4 Identification of virulence genes

#### 3.4.1 Cag Pathogenicity Island and *CagA* genotype

Ninety percent (18/20) of the isolate genomes harbored a complete CagPAI. Of these, ten CagPAI were associated with HpAsia2 lineage isolates and eight belonged to HpEurope lineage isolates. All CagPAI of HpAsia2 lineage isolates were lacking both DNA and DNA‐RNA helicases coding sequences while five of the HpEurope lineage isolates were missing only DNA helicase when compared with Western type CagPAI sequence of 26695 strain (Figure S2A). Comparison of CagPAI sequences with East Asian strain (F57) showed that six isolates (five HpAsia2 and one HpEurope) manifested a major gap of 2093 kb which encodes for a hypothetical protein in F57 (Figure S2B).

Virulence factor mapping indicates most of the Bangladesh isolates harbored 118 to 131 virulence genes with the exception of two (61A5, 152B5; CagPAI‐negative strain lacking *cag1* to *cag5* and *cagA* to *cagZ*) (Figure S3). Moreover, all urease enzymes, most of the flagellar associated proteins, endotoxins, and most of the Lewis antigens such as FutA, FutB, and NeuA/FlmD were found in most of the isolates (Figure S3).

**Figure 2:**
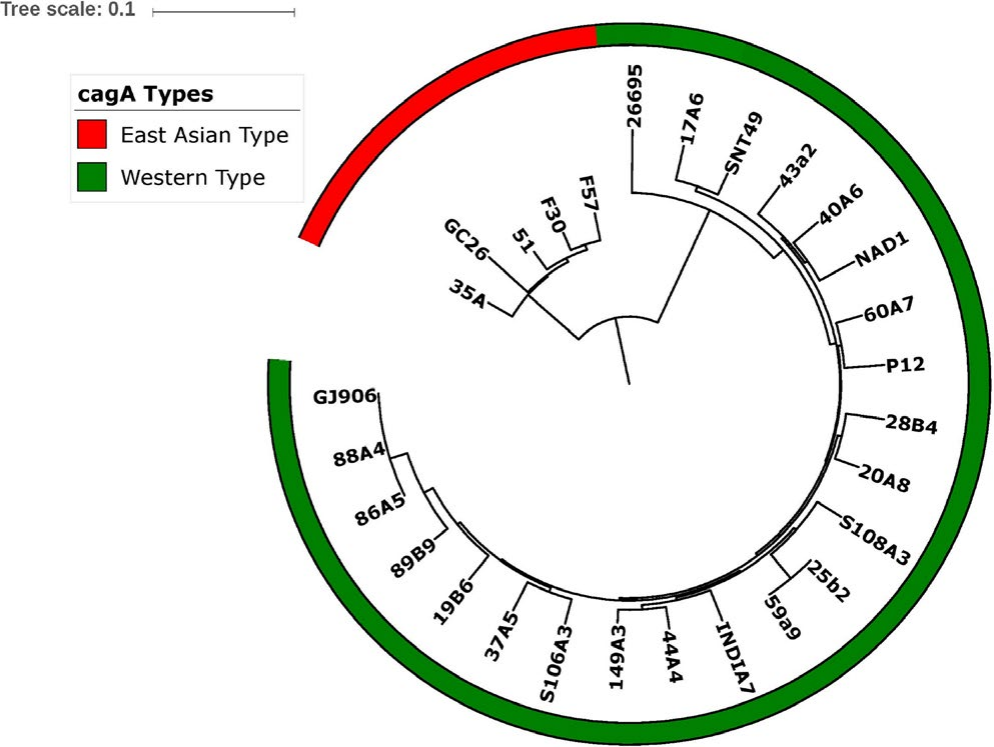
The *cagA* gene‐based phylogenetic tree of 20 BD *H pylori* isolates generated using Mega 5.2 with a bootstrap replicates of 1000 employing neighbor‐joining (NJ) algorithm and visualized in iTOL (A)

The phylogenetic analysis of *cagA* gene of 18 Bangladeshi *H pylori* isolates together with five Western and five East Asian *cagA* gene sequences, revealed a close clustering with the Western strains and not with East Asian strains as shown in Figure 2. In all Western type *cagA*, 55.5% (10/18) of the population were clustered with HpAsia2 while 44.45% with HpEurope (Figure 1). A closer examination of these gene sequences revealed AB‐C EPIYA motif in 15 (75%) isolates which is more reflective of HpEurope lineage. Two (10%) isolates had one extra EPIYA‐C motif (ABC‐C type) as compared to normal AB‐C EPIYA motif in which one was associated with HpAsia2 and other with HpEurope. Moreover, one isolate belonged to HpEurope was lacking EPIYA‐(C) motif (AB type) (Table 2). Seven *cagA*‐encoded strains (six AB‐C type and one ABC‐C) possessed an EPIY (T) B‐type motif while remaining isolates (9 AB‐C, 1 ABC‐C, and 1 AB types) contained an EPIY (A) B‐type motif at C‐terminal. In addition to this, we found that 5 out of 7 EPIY (T) B‐type motif harboring isolates were associated to HpAsia2 (25b2, 59a9, 86A5, 88A4, and GJ906) while only 2 belonged to HpEurope (17A6 and S108A3) (Figure 1).

**Table 2:** Prevalence of *Helicobacter pylori* virulence factors (*cagA* and *vacA*) among different lineage of isolates

Genotypes description	Total, no. (%)	Lineage wise distribution	
		HpAsia2	HpEurope
Total studied	20	10 (50%)	10 (50%)
*cagA*			
*cagA positive*	18 (90)	10 (55.5%)	8 (44.45%)
AB	1 (5)	0 (0%)	1 (100%)
ABC	15 (75)	9 (60%)	6 (40%)
ABCC	2 (10)	1 (50%)	1 (50%)
*cagA negative*	2 (10)	0 (0%)	2 (100%)
Pre‐EPIYA type (no deletion)	18 (100)	10 (55.5%)	8 (44.45%)
*vacA*			
s1	18 (90)	10 (55.5%)	8 (44.45%)
s2	2 (10)	0 (0%)	2 (100%)
m1	15 (75)	8 (53.3%)	7 (46.6%)
m2	5 (25)	2 (40%)	3 (60%)
i1	18 (90)	10 (55.5%)	8 (44.45%)
i2	2 (10)	2 (100%)	0 (0%)
d1	18 (90)	10 (55.5%)	8 (44.45%)
d2	2 (10)	2 (100%)	0 (0%)
c1	15 (75)	8 (53.3%)	7 (46.6%)
c2	5 (25)	2 (40%)	3 (60%)
*vacA* s1m1i1d1c1	15 (75)	8 (53.3%)	7 (46.6%)
s1m2i1d1c2	3 (15)	2 (66.6%)	1 (33.3%)
s2m2i2d2c2	2 (10)	0 (0%)	2(100%)
*CagA* positive/*vacA* s1m1i1d1c1	15 (75)	8 (53.3%)	7 (46.6%)
s1m2i1d1c2	3 (15)	2 (66.6%)	1 (33.3%)
*CagA* negative/*vacA* s2m2i2d2c2	2 (10)	0 (0%)	2(100%)

Moreover, sequence analysis of the pre‐EPIYA revealed that none of the isolates had a 39 bp deletion (approximately 300‐bps upstream of the first EPIYA motif) that is typically observed in strains from Western countries. A total 55 EPIYA motifs were identified in 18 *cagA*‐positive isolates. We identified two types of motifs: EPIY (A) (48/55, 87.2%) and EPIY (T) (7/55, 12.7%). The EPIYA‐B contained two types of motifs (EPIYA, EPIYT), and EPIY (T) was found in this motif (Table S3).

#### 3.4.2 *VacA* Genotype

**Figure 3:**
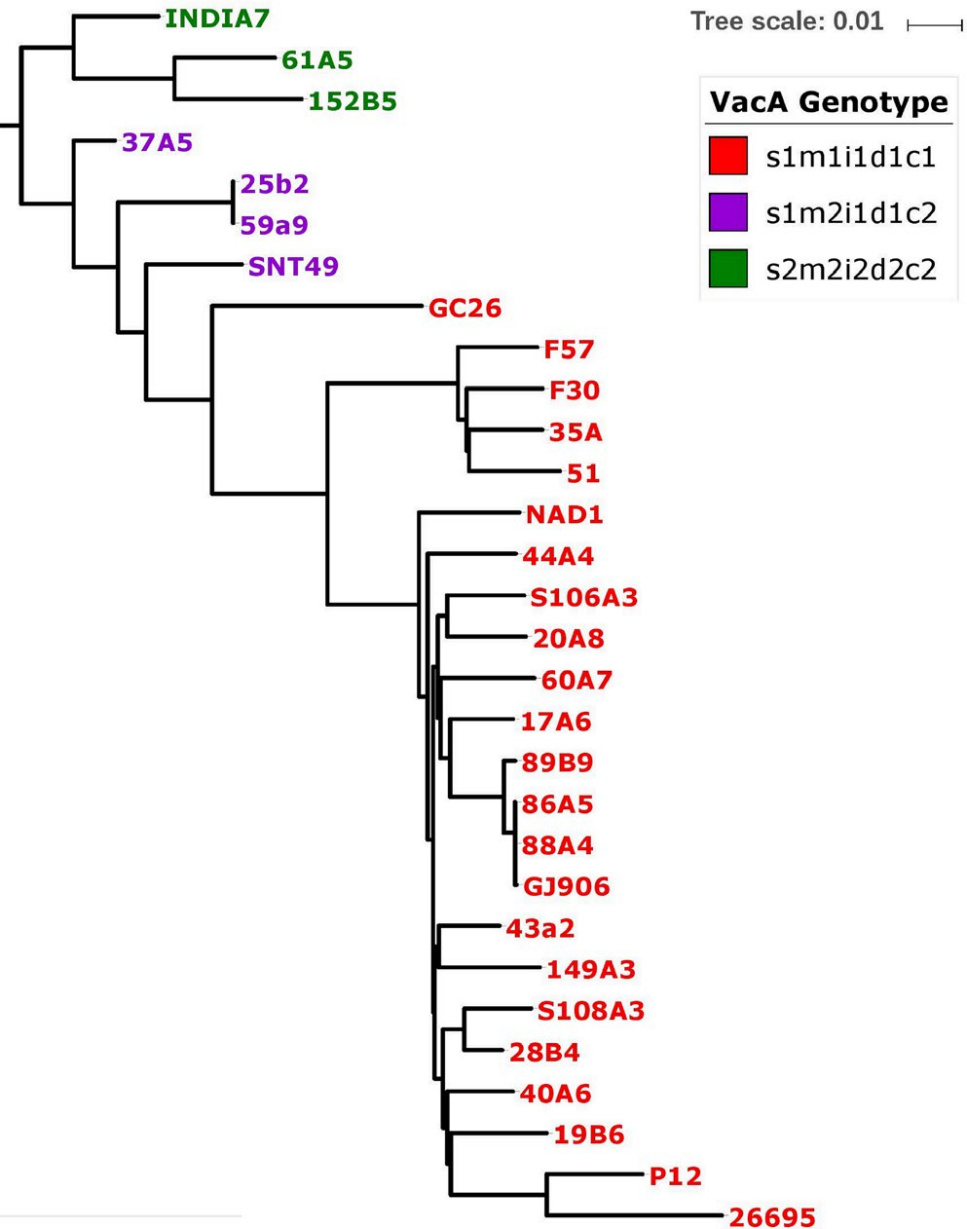
The *vacA* gene‐based phylogenetic tree of 20 studied *H pylori* strains generated using Mega 5.2 with a bootstrap replicates of 1000 employing neighbor‐joining (NJ) algorithm and visualized in iTOL (A)

The predominant *vacA* “s” allele type was s1 (18/20, 90%) which is a typical of HpAsian and Western strains. The remaining isolates were s2 genotype and were present in HpEurope lineage isolates. The occurrence rate of the *vacA* m1 allele type was 75% (15/20) while the m2 allele type accounted for 25% (5/20). *VacA* gene‐based phylogenetic analysis of all 20 Bangladeshi isolates together with ten Western and East Asian harboring *vacA* gene revealed a clear distinction between m1 and m2 sequences (Figure 3). The prevalence of s1m1 genotype was found to be 75% (15/20), while 15% was s1m2 and 10% s2m2 in all 20 isolates. An examination of allelic diversity of different regions of *vacA* genotype found that all isolates that possessed s1 and m1, also retained i1, d1, and c1 (75%, 15/20). In contrast, all isolates that contained less‐virulent genotype s2 and m2 harbored i2, d2, and c2 genotype (10%, 2/20). Moreover, both *cagA*‐ and *vacA*‐positive isolates were predominant with genotype s1m1i1d1c1 while other group of less‐virulent isolates contained s1m2i1d1c1 genotype. A third *cagA*‐negative and non‐virulent group was identified as s2m2i2d2c2 genotype (Table 2).

### 3.5 Outer membrane proteins analysis

#### 3.5.1 *OipA* gene

We found that *oipA* gene is present in all 20 isolates and showed five different CT repeat patterns (Table 3). Furthermore, we found that nine isolates out of 20 contained six CT repeat sequence displaying the “ON” status of *OipA*, while five strains that also had similar status (“ON”) contained (2 + 3) pattern of CT repeats. Two strains were found to harbor a CT repeat architecture of (5 + 2) and another two with nine direct repeat of CT dinucleotide and in both cases the gene status was “ON.” In addition to this, 61A5 and 89B9 isolates were having 10 direct CT dinucleotide repeat and in that case gene status was found to be “OFF” and these two were belonged to HpEurope lineage.

**Table 3:** Number and types of repeats and “On and Off” status of *OipA* gene in 20 *H pylori* strains

Strains	Number and types of repeats	Status	Hp Lineage
149A3	6	On	HpAsia2
20A8	6	On	HpAsia2
25b2	6	On	HpAsia2
28B4	6	On	HpAsia2
37A5	6	On	HpEurope
43a2	6	On	HpEurope
59a9	6	On	HpAsia2
60A7	6	On	HpEurope
S108A3	6	On	HpEurope
17A6	2 + 3	On	HpEurope
86A5	2 + 3	On	HpAsia2
88A4	2 + 3	On	HpAsia2
152B5	2 + 3	On	HpEurope
GJ906	2 + 3	On	HpAsia2
44A4	5 + 2	On	HpAsia2
19B6	5 + 2	On	HpEurope
40A6	9	On	HpEurope
S106A3	9	On	HpAsia2
89B9	10	**Off**	HpEurope
61A5	10	**Off**	HpEurope

#### 3.5.2 *Bab* genes

The prevalence of *bab* genes among the isolates were studied. The *babA* gene was detected in 75% (15/20) of the isolates, while *babB* gene was identified in 55% (11/20) strains. The *babA* gene was found in all HpAsia2 population isolates, but was present in only 50% of HpEurope isolates. The genotype *babA*/*babB*/‐ (*babA* and *babB* present and *babC* absent) was detected in 40% (8/20) isolates, and six of these eight isolates were HpAsia2 isolates. We also observed that *babA* was absent in both s2m2i2d2c2 and one of the three s1m2i1d1c1 *vacA* genotype isolates (Figure S4). Clinical outcome data showed that isolates with (*babA*/*babB*/‐) or (*babA*/‐/‐), *cagA* positive and the s1m1i1d1c1 *vacA* genotypes were associated with either more or less severe clinical gastric outcome, while normal clinical outcomes were observed for (‐/*babB*/‐) or (‐/‐/‐) isolates except in 37A5 (Table S4).

### 3.6 In silico antibiotics susceptibility analysis

In silico antimicrobial susceptibility analysis revealed that 40% of the strains were multi‐drug resistant. We also detected that 90% (18/20) of the strains were resistant to metronidazole. Of those metronidazole‐resistant strains, nine were expected to express non‐functional or altered RdxA and/or FrxA proteins resulted from truncation, frameshift mutations, insertion of extra bases, and partial gene deletions. The remaining nine genotype‐based predicted metronidazole‐resistant strains resulted from amino acid exchanges (R16C, K64N, and P106S) that merely occur in the *rdxA* gene of metronidazole‐resistant *H pylori* strains. Of those nine strains, two exhibited double mutations (K64N and P106S) while one contained triple mutations (R16C, K64N, and P106S) (Table 4).

**Table 4:** Truncations, frameshift mutations, insertion, deletions, and amino acid exchanges identified in the *rdxA*, *frxA*, *gyrA*, and *23S rRNA* genes of sequenced *H pylori* isolates

Genes	Mutation type (Position)	No. of *H pylori* strain (s)	References
*rdxA*	Nonsense (50)	4	Truncations in *rdxA* suggested to lead to metronidazole résistance^23^
	Nonsense (86)	1	
	Fragmented	1	Fragmentation in *rdxA* suggested to be responsible for metronidazole résistance^31^
	Frameshift (146)	1	These frameshift, deletions have not been previously reported in the literature
	deletion of 35 bps N‐terminal region	1	
	deletion of 18 bps between codon 91 to 97 of N‐terminal region	1	
	R16C	1	R16C occurs only in metronidazole‐resistant *H pylori* strains^24^, ^25^, ^26^
	K64N	7	K64N occurs solely in metronidazole‐resistant *H pylori* strains^27^
	P106S	5	P106S occurs solely in metronidazole‐resistant *H pylori* strains^27^
*frxA*	Frameshift (18)	5	Frameshifts in *frxA* suggested to confer metronidazole resistance^31^
	Frameshift (106)	1	
	Nonsense (73)	2	This nonsense mutation has not been previously reported in the literature
	Insertion (45 543 bp)	1	This insertion in *frxA* has not been previously reported in the literature
*gyrA*	5‐ amino acid N‐terminal extension (MQDNS)	5	5‐amino acid N‐terminal extension suggested to confer fluoroquinolone resistance^31^
	D91G	2	Amino‐ acid exchanges in QRDR of *GyrA* suggested to confer resistance to fluoroquinolone^31^
	D91N	1	
	R295C	1	Amino acid exchange at 295 suggested to be responsible for fluoroquinolone resistant in *H pylori* ^31^
*23S rRNA*	G2224A	1	G2224A mutation in *23S rRNA* occurs solely in clarithromycin‐resistant *H pylori* strain^31^

An extraordinary N‐terminal extension of GyrA by five amino acid residues (QDNSV) and amino acid exchanges in QRDR (N87, D91, and R295) occurs solely in fluoroquinolone‐resistant *H pylori*.^31^ Our genotypic analysis also revealed that 45% (9/20) of the *H pylori* strains were resistant to fluoroquinolone antibiotics. Out of these nine fluoroquinolone‐resistant strains, five had an unprecedented N‐terminal extension of GyrA by five amino acid residues, immediately after starting codon, three exhibited amino acid exchanges in QRDR (D91) of *H pylori* and one strain showed a mutation at R295. Point mutation at G2224 in *23S rRNA* was detected in only one strain which is suggested to confer resistance to clarithromycin (Table 4).

## 4 DISCUSSION

*Helicobacter pylori* has a complex and long‐standing coexistence with humans, so much so that particular bacterial lineages are strongly associated with regionally associated human lineages.^8^ The plasticity of the *H pylori* genome, in particular recombination leads to situations in a modern world, where human populations are more mobile, where the long‐standing patterns of co‐evolution are uncoupled with instances of adverse clinical consequences for the host.^50^ Hence, identifying the demographic composition and understanding the virulence potential of the region's dominant strains becomes essential. Here, we sequenced 20 Bangladeshi *H pylori* isolates and performed whole genome–based comparative analysis to investigate and understand the genetic architecture and virulence gene profile of these isolates.

*Helicobacter pylori* can be categorized into seven distinct lineages with names that are associated with geographic regions but are really representative of the human lineages that have been long‐term inhabitants of these regions: HpAfrica1, HpAfrica2, HpSahul, HpEurope, HpAsia2, HpAmerind, and HpEastAsia.^8^, ^10^ In this study, the 20 isolates from one ethnic group, namely Bengali, were not uniform in their *H pylori* lineage. The observation of two distinct lineages has been also reported previously.^51^ The maintenance of independent lineages of *H pylori* within a stable ethnic group seems counterintuitive given the plasticity of the *H pylori* genome and an expectation that these co‐existent lineages would converge over time. The *H pylori* lineages present are consistent being at a point of intersection between humans co‐evolving with HpAsia2 lineage *H pylori* on the Indian subcontinent and humans from Central Asia with ancestral links to Europe. The mobility of ancient human populations is highlighted by *H pylori* genome from a European Copper age glacier mummy found in the European Alps belonged to HpAsia2 lineage.^52^ Other studies, however, investigated that all Indian strains sequenced from indigenous Indians, who are predominantly Aryan and Dravidian ancestry, have significant homology to the HpEurope population suggested that introduction of *H pylori* took place with Indo‐Aryan migration.^53^

The accessory genome content provides an overview of differences in gene content between Bangladeshi isolates, while the core genome comparison has allowed to observe the generalized preservation of lineage‐specific differences. Higher percentage of core genes belonging to J (translation, ribosomal structure, and biogenesis) and M (cell membrane/biogenesis envelope) functional classes also identified in our analysis may potentially indicate the adaptive stress imposed by the dynamic micro‐environment of the stomach to survive on this gastric pathogen as also previously reported.^43^ Analysis of this study also revealed that a majority of specific genes belonging to L (replication, recombination, and repair) functional classes also indicating the requirement of *H pylori* to maintain a robust recombination and repair mechanism.

Based on the incidence rate of gastric cancer in people from Europe and South‐Central Asia, HpEurope population is thought to be associated with higher gastric cancer risk than HpAsia2 (http://globocan.iarc.fr). An unexpected result from our study which revealed that all HpAsia2 strains had *cagA* gene while two HpEurope isolates were completely lacking *cagA gene*, even the whole CagPAI, suggesting that HpAsia2 strains may be more virulent than HpEurope isolates. This study also corroborate with a similar type of comparative genomic study of *H pylori* isolates from Bangladesh, which showed that subjects infected with HpAsia2 had greater activity and inflammation in the antrum than with patients where HpEurope isolates were obtained which might be due to higher proportion of less‐virulent *cagA* and *vacA* genotype in HpEurope isolates.^51^ In corroboration with this, *oipA* gene analysis result of this study identified that expression status of *oipA* gene of all the isolates (100%) of HpAsia2 was “ON,” while 20% of the HpEurope isolates were “OFF.” This result also suggested that the inducing potential of inflammation in HpAsia2 strains is high as compared to HpEurope.

There are several reports about the association of BabA‐positive status of *H pylori* with increased risk for the development of peptic ulcer disease.^21^ In this study, we looked into the prevalence of different *bab* genes in various combination among the isolates. The study revealed that all HpAsia2 isolates were harboring *babA* and also most of the isolates had both *babA* and *babB* and no *babC* gene (*babA*/*babB*/‐). Clinical outcomes of the patients carrying HpAsia2 lineage *H pylori* showed more gastric severity as compared to HpEurope carriers. This could be one of the important explanation because of absence of *babA* or *babA*/*babB*/‐ genes in most of the Bangladeshi isolates of HpEurope lineage. This result is also in concordance with the others which explains that the pathogenic potential of HpAsia2 is more as compared to HpEurope as described above.

Prevalence of metronidazole‐resistant *H pylori* in Bangladesh is quite high, with resistance rate more than 90%. The resistance rates of levofloxacin and clarithromycin have been also increasing in *H pylori* isolates of Bangladesh.^32^, ^54^ Although the main mechanism of acquiring metronidazole resistance involves RdxA and/or FrxA inactivation mutations, a considerable number of missense mutations in both *rdxA* and *frxA* genes cannot rule out their role in metronidazole resistance by inducing conformational changes of RdxA and FrxA proteins. In tandem with remarkably high occurrence of metronidazole, our study also showed that about half of the strains would be resistant to fluoroquinolones. This acquired resistance is primarily due to amino acid substitution mutations in QRDR of GyrA protein that we also observed in some genotype predicted fluoroquinolone‐resistant strains. Interestingly, more than half of the predicted fluoroquinolone‐resistant strains had an insertion of QDNSV residues immediate to the start codon at N‐terminal region. This insertion of five amino acid residues would likely cause a conformational change in the GyrA protein and thus reducing its binding affinity to fluoroquinolone antibiotics. Importantly, genotype‐based analysis of antibiotic susceptibility of our study showed a high occurrence of metronidazole‐ and fluoroquinolone‐resistant *H pylori* strains in Bangladesh. This suggests that triple therapy based on metronidazole and fluoroquinolone may not be useful to treat *H pylori* infection in Bangladesh.

In conclusion, we showed that Bangladeshi strains with similar antibiotic resistance pattern, separated into two major population with distinct *cagA* and *vacA* genotypes. The comparative analysis of genomes provides important clues to the virulence potential of two segregated population (HpAsia2 and HpEurope). The study revealed that HpAsia2 strains are more virulent as compared to HpEurope in Bangladesh. Finally, as the pathology of *H pylori* is multifactorial, it would be important to understand even host and environmental factors in addition to bacterial genotype to better understand the disease outcome.

Our study had several limitations; a two‐step randomization procedure used in this study may not represent all variations which might be available in a population. In silico analysis of antimicrobial resistance may not identify novel or yet to be determined mutations associated with antimicrobial resistance.

## DISCLOSURE

The authors have no competing interests.

## Supporting information

Fig S1A

Fig S1B

Fig S1C

Fig S2A

Fig S2B

Fig S3

Supplementary Material

Table S1

Table S2

Table S3

Table S4

Supplementary Material
