# Supplementary figures and images for: A comparative whole genome analysis of Helicobacter pylori from a human dense South Asian setting
Source: Helicobacter. 2020 Oct 18;26(1):e12766. doi: 10.1111/hel.12766 (PMC7816255; doi:10.1111/hel.12766)

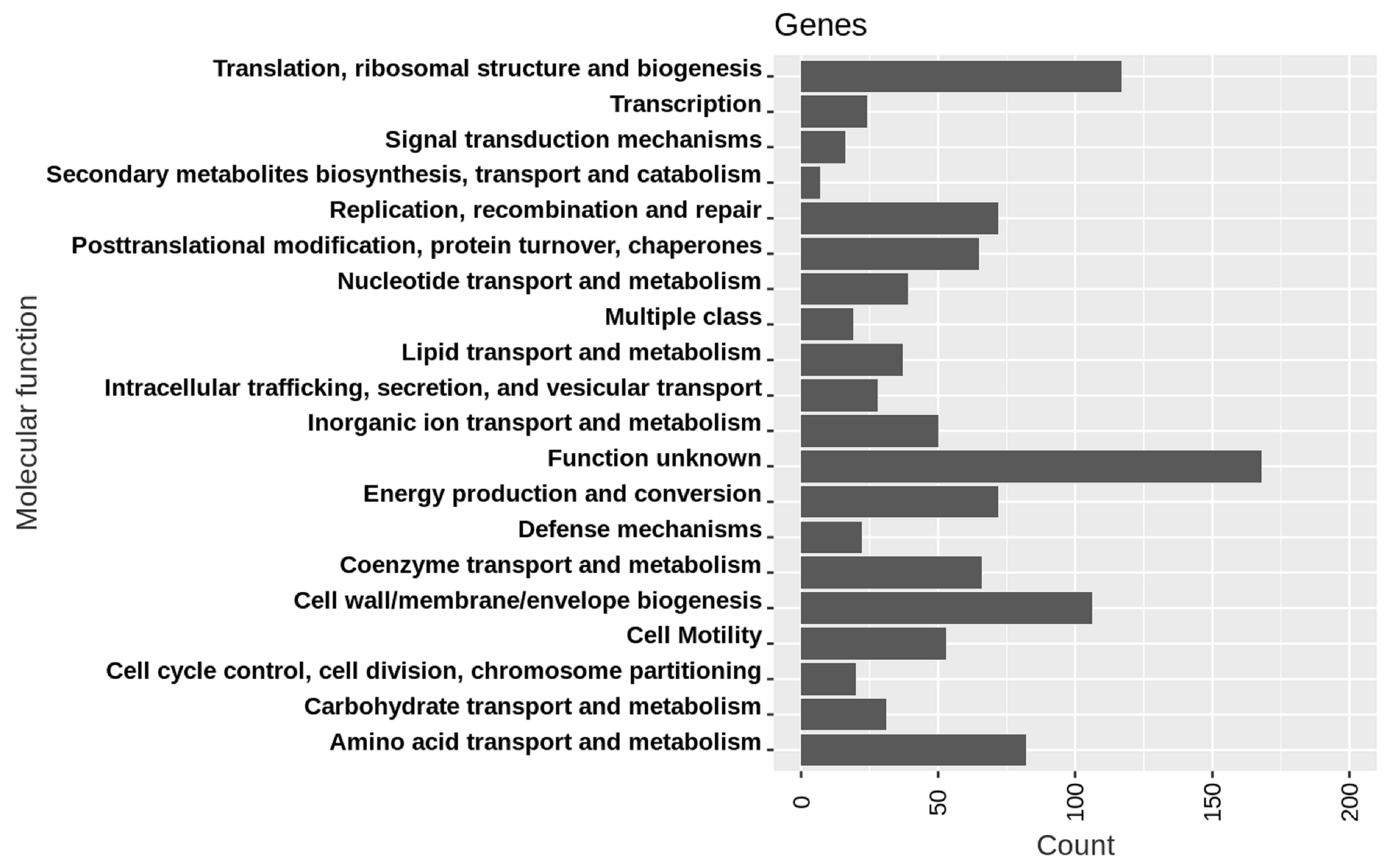

Supplement: Supplementary file 1 — Fig S1A [file HEL-26-e12766-s001.tif]

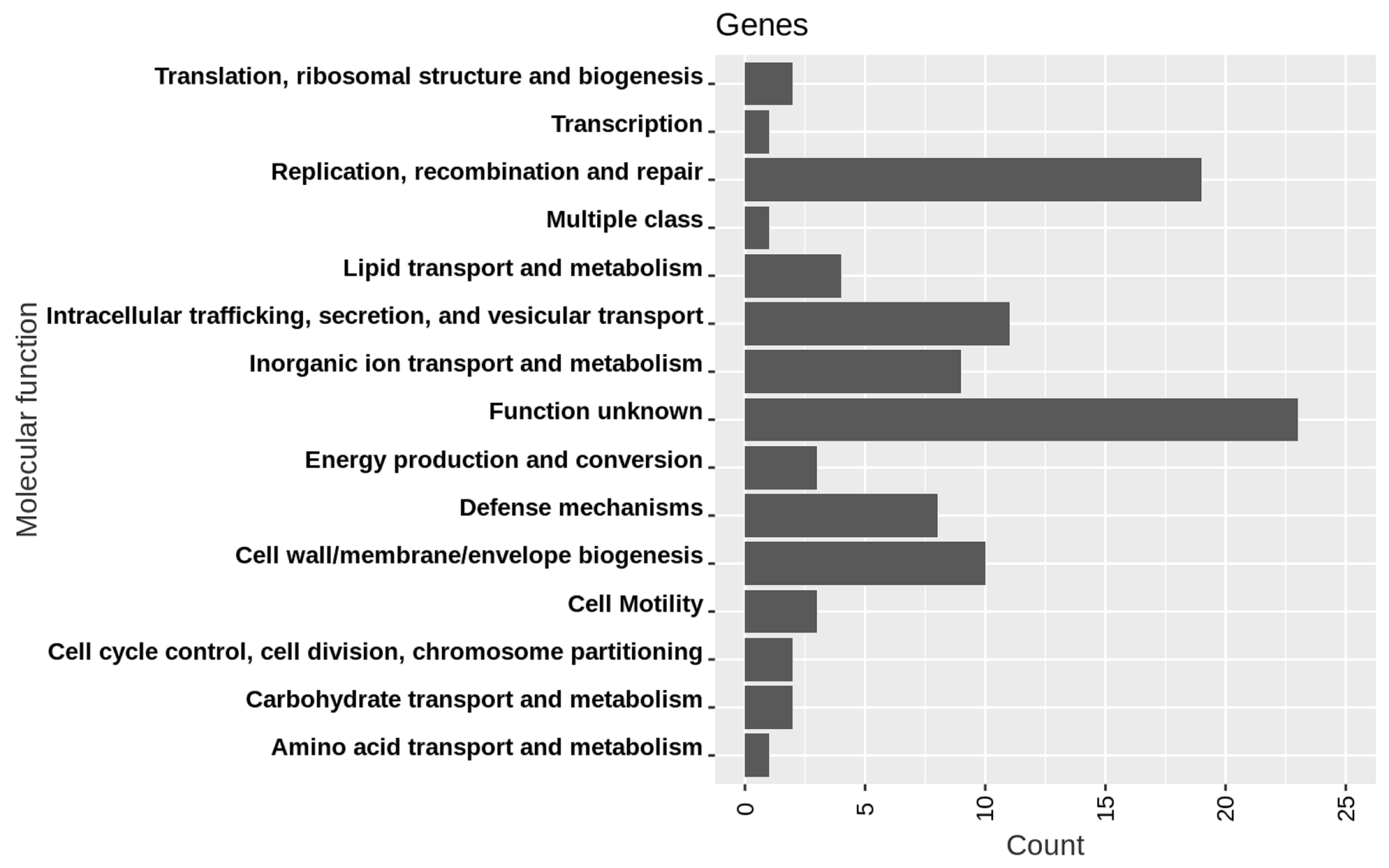

Supplement: Supplementary file 2 — Fig S1B [file HEL-26-e12766-s002.tif]

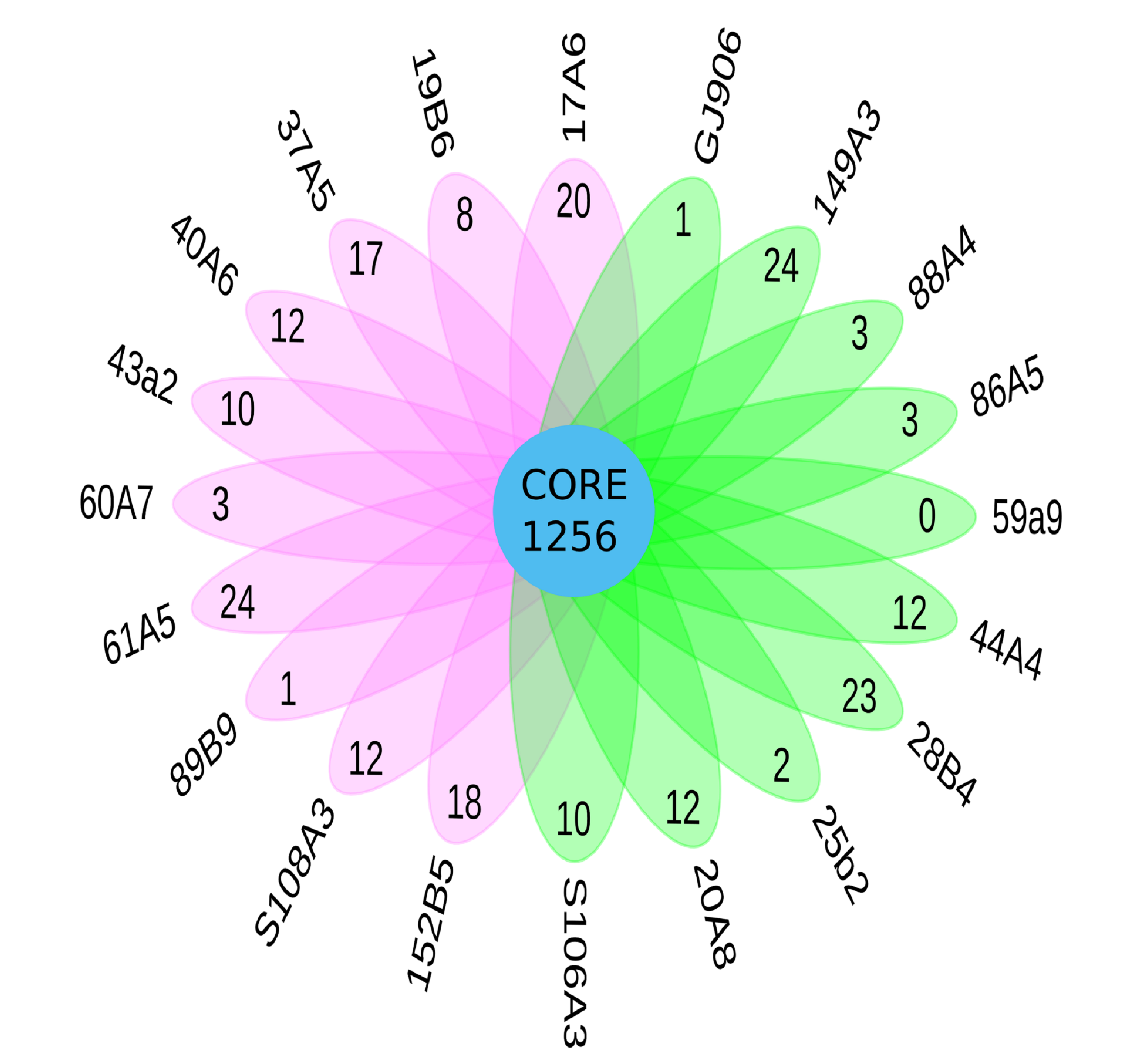

Supplement: Supplementary file 3 — Fig S1C [file HEL-26-e12766-s003.tif]

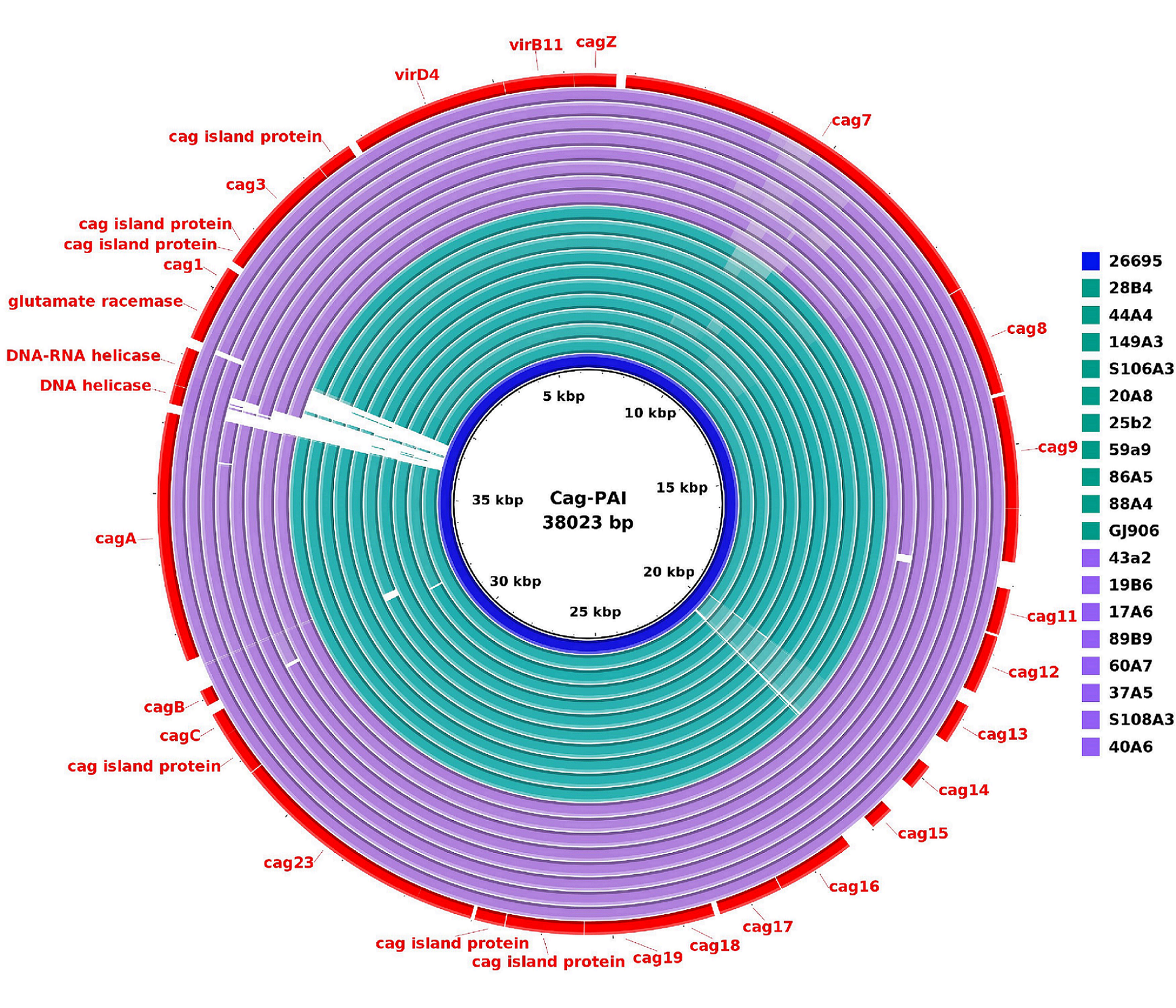

Supplement: Supplementary file 4 — Fig S2A [file HEL-26-e12766-s004.tif]

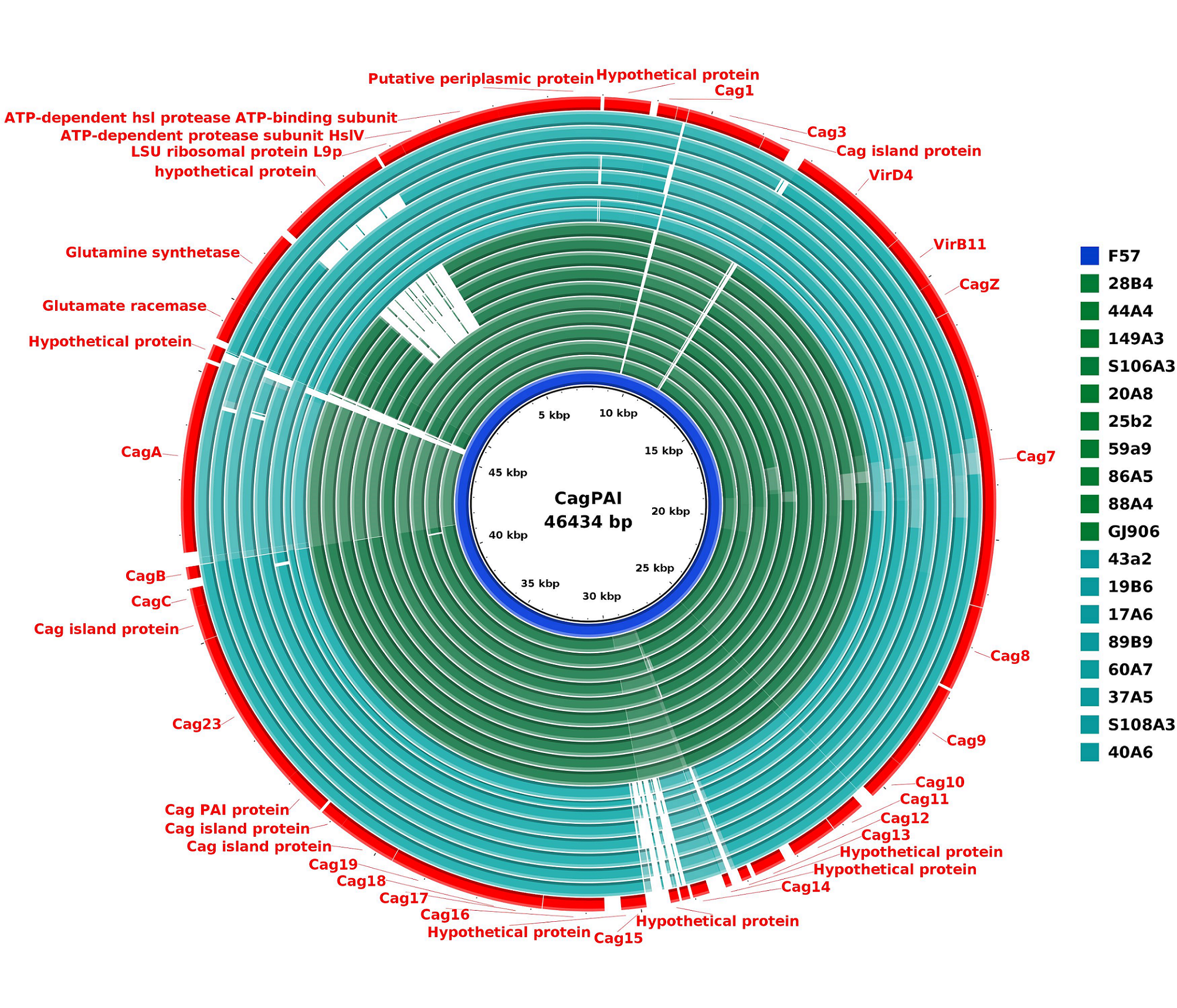

Supplement: Supplementary file 5 — Fig S2B [file HEL-26-e12766-s005.tif]

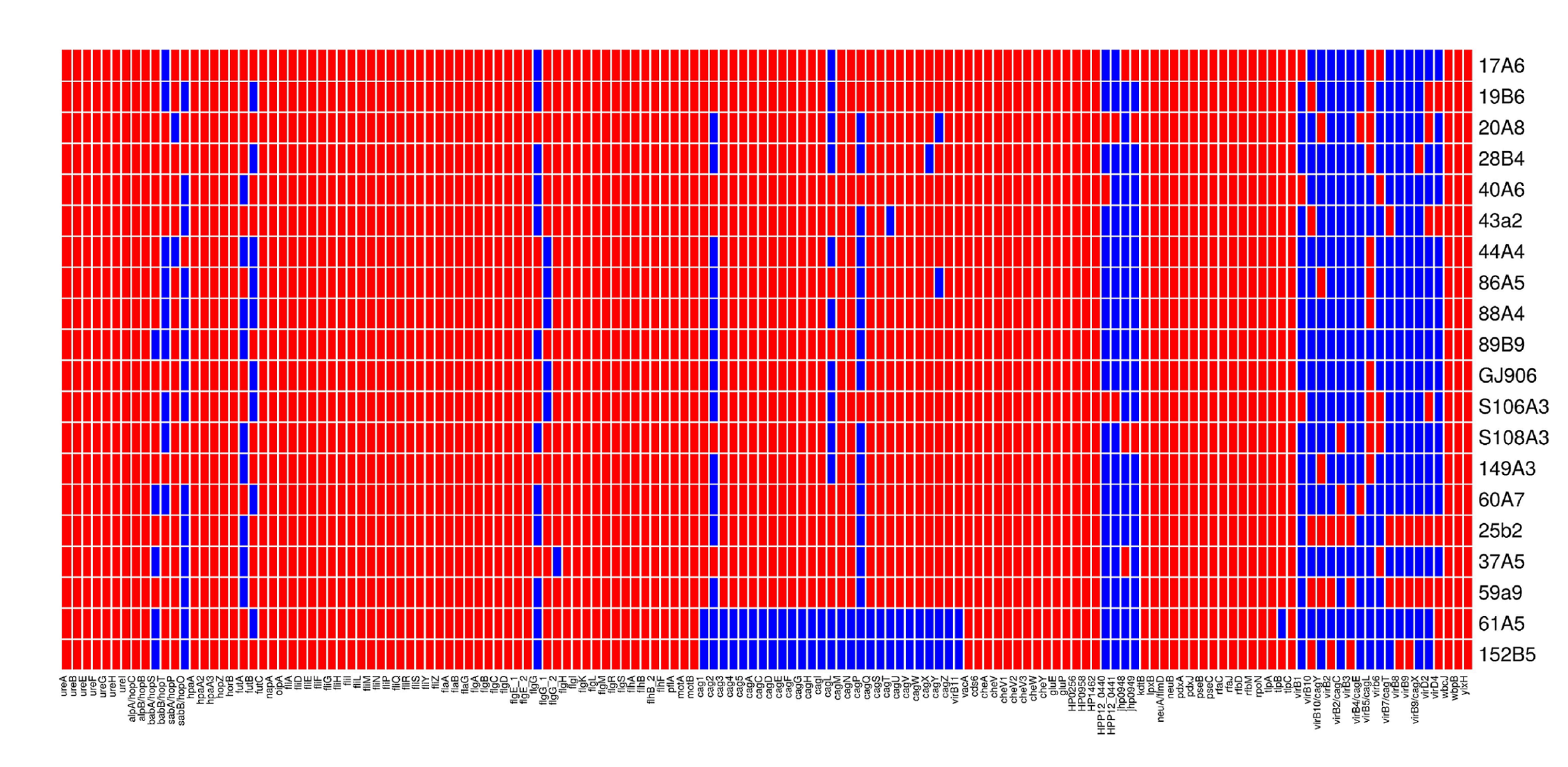

Supplement: Supplementary file 6 — Fig S3 [file HEL-26-e12766-s006.tif]

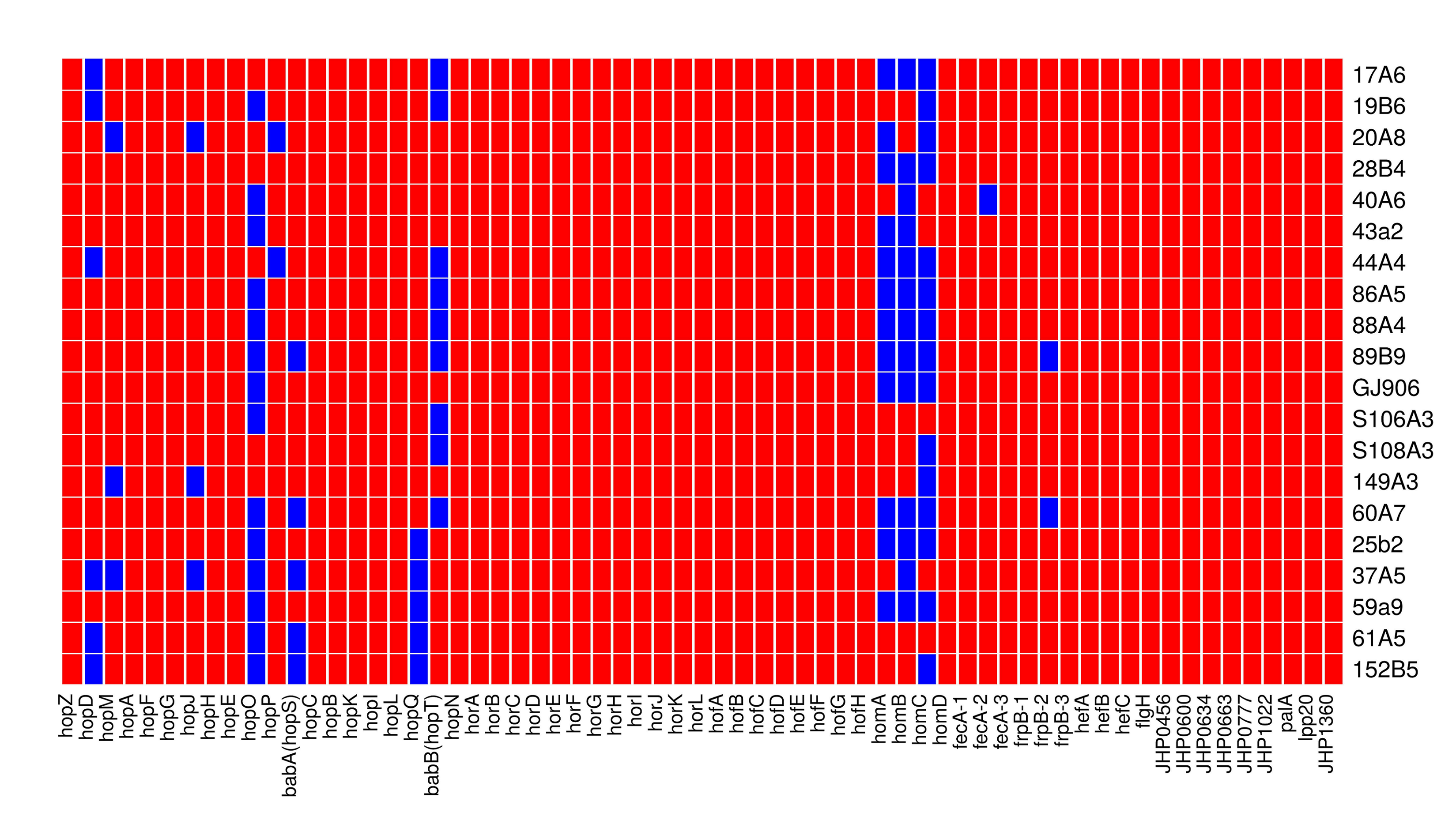

Supplement: Supplementary file 7 — Supplementary Material [file HEL-26-e12766-s007.tif]
