# Supplementary material for: A comparative whole genome analysis of Helicobacter pylori from a human dense South Asian setting
Source: Helicobacter. 2020 Oct 18;26(1):e12766. doi: 10.1111/hel.12766 (PMC7816255; doi:10.1111/hel.12766)
Supplement: Supplementary file 8 — Table S1 [file HEL-26-e12766-s008.docx]

| **No.** | **Strain ID** | **Accession Number** | **Isolated from** |
| --- | --- | --- | --- |
| 1 | **SouthAfrica50** | NZ_AVNI00000000 | South Africa |
| 2 | **SouthAfrica 20** | NC_022130 | South Africa |
| 3 | **SouthAfrica07** | NC_017361 | South Africa |
| 4 | **J99** | NC_000921 | USA |
| 5 | **908** | NC_017357 | France |
| 6 | **NAD1** | NZ_AJGJ00000000 | India |
| 7 | **26695** | NC_000915 | UK |
| 8 | **B8** | NC_014256 | USA |
| 9 | **HPAG1** | NC_008086 | Sweden |
| 10 | **P12** | NC_011498 | German |
| 11 | **SNT49** | NC_017376 | India |
| 12 | **INDIA7** | NC_017372 | India |
| 13 | **NAK7** | NZ_AONJ00000000 | India |
| 14 | **NAB47** | NZ_AJFA00000000 | India |
| 15 | **F32** | NC_017366 | Japan |
| 16 | **V225d** | NC_017355 | Venezuela |
| 17 | **SHI112** | NC_017741 | Peru |
| 18 | **Cuz20** | NC_017358 | Peru |
| 19 | **SAT464** | NC_017359 | Peru |
| 20 | **SHI470** | NC_010698 | Peru |
| 21 | **SHI169** | NC_017740 | Peru |
| 22 | **SHI417** | NC_017739 | Peru |
| 23 | **83** | NC_017375 | Japan |
| 24 | **F16** | NC_017368 | Japan |
| 25 | **OK310** | NC_020509 | Japan |
| 26 | **F57** | NC_017367 | Japan |
| 27 | **GC26** | NZ_AKHV00000000 | Malaysia |
| 28 | **OK113** | NC_020508 | Japan |
| 29 | **51** | NC_017382 | Korea |
| 30 | **F30** | NC_017365 | Japan |
| 31 | **35A** | NC_017360 | USA |
| 32 | **17A6** | JABKCC000000000 | Bangladesh |
| 33 | **19B6** | JABKCD000000000 | Bangladesh |
| 34 | **25b2** | JABKCE000000000 | Bangladesh |
| 35 | **20A8** | JABKCF000000000 | Bangladesh |
| 36 | **28B4** | JABKCG000000000 | Bangladesh |
| 37 | **37A5** | JABKCH000000000 | Bangladesh |
| 38 | **40A6** | JABKCI000000000 | Bangladesh |
| 39 | **43a2** | JABKCJ000000000 | Bangladesh |
| 40 | **44A4** | JABKCK000000000 | Bangladesh |
| 41 | **59a9** | JABKCL000000000 | Bangladesh |
| 42 | **60A7** | JABKCM000000000 | Bangladesh |
| 43 | **61A5** | JABKCN000000000 | Bangladesh |
| 44 | **149A3** | JABKCO000000000 | Bangladesh |
| 45 | **86A5** | JABKCP000000000 | Bangladesh |
| 46 | **88A4** | JABKCQ000000000 | Bangladesh |
| 47 | **89B9** | JABKCR000000000 | Bangladesh |
| 48 | **GJ906** | JABKCS000000000 | Bangladesh |
| 49 | **S106A3** | JABKCT000000000 | Bangladesh |
| 50 | **S108A3** | JABKCU000000000 | Bangladesh |
| 51 | **152B5** | JABKCV000000000 | Bangladesh |
