# Supplementary material for: A comparative whole genome analysis of Helicobacter pylori from a human dense South Asian setting
Source: Helicobacter. 2020 Oct 18;26(1):e12766. doi: 10.1111/hel.12766 (PMC7816255; doi:10.1111/hel.12766)
Supplement: Supplementary file 9 — Table S2 [file HEL-26-e12766-s009.docx]

S Table 2. Putative phage regions identified using PHASTER tool and major genes encoded within these regions.

| **Strains** | **Regions** | **Size (kb)** | **CDS** | **Completeness** | **Major functions encoded** |
| --- | --- | --- | --- | --- | --- |
| 17A6 | 1 | 34.3 | 41 | Complete | Hypothetical proteins, terminase,portal,tail fiber,transposase,integrase |
| 19B6 | 1 | 21.1 | 12 | Incomplete | Hypotehtical proteins, head,transposase |
| 28B4 | 1 | 52.6 | 32 | Complete | Hypotheticalproteins,terminase,portal, |
|  |  |  |  |  | integrase, putative primase, DNA helicase |
| 37A5 | 1 | 32.1 | 39 | Complete | Hypotehtical proteins, terminase, integrase,DNA helicase, structrual proteins, tail fibers, primase, tail assembly, DNA repair |
| 43a2 | 1 | 17.3 | 12 | Incomplete | Hypothetical proteins,PRGA formyltransferase,DNA methylase, transposase,antitoxin HicB |
|  |  |  |  |  | IS element Dka2 orfB |
| S106A3 | 1 | 17.8 | 23 | Complete | Hypothetical proteins, terminase, portal proteins, putative holin, structrual proteins |
| S108A3 | 1 | 22.7 | 17 | Incomplete | Hypotehtical proteins, primase, DNA repair proteins, phage replication proteins, putative transcriptional regulator, integrase, recombinase, ABC transporter,DnaX DNA polymerase III clamp loader complex gamma-tau-delta subunit |
|  | 2 | 19.7 | 9 | Incomplete | Hypotehtical proteins, antitoxin HicB, transposase, DNA methylase, |
| 20A8 | 1 | 16 | 21 | Questionalble | Hypotehtical proteins, putative portal, structrual proteins, JHP1044-like mosaic proteins |
|  | 2 | 12.8 | 18 | Questionalble | Hypotehtical proteins, structrual proteins |
| 149A3 | 1 | 31.2 | 23 | Questionalble | Hypotehtical proteins, DNA helicase, integrase, terminase, primase, DNA repair |
| 61A5 | 1 | 18.5 | 28 | Questionalble | Hypotehtical proteins, GTPase, DNA replication,tail fiber, transposase, structrual proteins, tail assembly, putative holin |
| 88A4 | 1 | 4.8 | 7 | Incomplete | Hypothetical proteins, JHP1044 mosaic endodeoxyribonuclease |
