# Supplementary material for: A comparative whole genome analysis of Helicobacter pylori from a human dense South Asian setting
Source: Helicobacter. 2020 Oct 18;26(1):e12766. doi: 10.1111/hel.12766 (PMC7816255; doi:10.1111/hel.12766)
Supplement: Supplementary file 10 — Table S3 [file HEL-26-e12766-s010.doc]

**Supplementary Table 3. Frequencies of EPIYA motifs in studied strains.**

| **All motifs** | **No.** | **A motif** | **No.** | **B motif** | **No.** | **C motif** | **No.** |
| --- | --- | --- | --- | --- | --- | --- | --- |
| EPIYA | 48 | EPIYA | 18 | EPIYA | 11 | EPIYA | 19 |
| EPIYT | 7 | - | - | EPIYT | 7 | - | - |
| Total | 55 | - | 18 | - | 18 | - | 19 |
