# Supplementary material for: A comparative whole genome analysis of Helicobacter pylori from a human dense South Asian setting
Source: Helicobacter. 2020 Oct 18;26(1):e12766. doi: 10.1111/hel.12766 (PMC7816255; doi:10.1111/hel.12766)
Supplement: Supplementary file 11 — Table S4 [file HEL-26-e12766-s011.doc]

**Supplementary table 4: Prevalence of *bab*** genes in all studied isolates with their clinical outcome descriptions.

| **Strains** | ***bab*** **genotypes** | **Hp Lineages** | **Description of clinical outcomes** |
| --- | --- | --- | --- |
| 17A6 | *babA+* | HpEurope | mild antral gastritis |
| 19B6 | *babA+* | HpEurope | mild oesophagitis with antral gastritis with erosive duodenitis |
| 44A4 | *babA+* | HpAsia2 | antral gastritis |
| 86A5 | *babA+* | HpAsia2 | mild oesophagitis with erosive antral gastritis with erosive duodenitis |
| 88A4 | *babA+* | HpAsia2 | mild antral gastritis |
| S106A3 | *babA+* | HpAsia2 | Erosive duodenitis e-antral gastritis e-oesophagitis |
| S108A3 | *babA+* | HpEurope | Erosive duodenitis e-antral gastritis |
| 25b2 | *babA+/babB+* | HpAsia2 | mild oesophagitis with mild antral gastritis |
| 28B4 | *babA+/babB+* | HpAsia2 | oesophagitis with erosive antral gastritis with erosive duodenitis |
| 20A8 | *babA+/ babB+* | HpAsia2 | mild antral gastritis |
| 40A6 | *babA+/babB+* | HpEurope | mild oesophagitis with erosive antral gastritis |
| 43a2 | *babA+/ babB+* | HpEurope | mild antral gastritis |
| GJ906 | *babA+/babB+* | HpAsia2 | not applicable |
| 59a9 | *babA+/babB+* | HpAsia2 | mild antral gastritis |
| 149A3 | *babA+/ babB+* | HpAsia2 | Erosive duodenitis e-erosive antral gastritis e- oesophagitis |
| 37A5 | *babB* | HpEurope | mild oesophagitis with erosive antral gastritis with erosive duodenitis |
| 152B5 | *babB* | HpEurope | Superficial antral gastritis. |
| 61A5 | *babB* | HpEurope | not applicable |
| 60A7 | *babA-/babB-* | HpEurope | normal findings |
| 89B9 | *babA-/babB-* | HpEurope | normal findings |
