# Supplementary material for: A comparative whole genome analysis of Helicobacter pylori from a human dense South Asian setting
Source: Helicobacter. 2020 Oct 18;26(1):e12766. doi: 10.1111/hel.12766 (PMC7816255; doi:10.1111/hel.12766)
Supplement: Supplementary file 12 — Supplementary Material [file HEL-26-e12766-s012.docx]

**S file 1:** Patient population, *H. pylori* isolation, culture of “Genetic diversity of *Helicobacter pylori* in colonization and infection: role in disease outcome (symptomatic and asymptomatic) and transmission” study and selection of strains for the present study.

525 Families in the database

112 randomly selected families invited

Inclusion & exclusion

55 families were eligible (312 members)

Stool antigen test

44 families were stool antigen positive (279 member)

174 agreed for endoscopy

(125 adult and 49 children)

275 samples for culture;

Adult 226 (117 antrum, 109 body); Children 49 (gastric juice)

105 refused endoscopy

(62 adult and 43 children)

Treated for *H. pylori*

(excluded from analysis)

201 samples (94 antrum, 83 body and 24 gastric juice) from 128 patient (125 adult and 24 children) were positive

Treated for *H. pylori* & data analysis

201x10=2010 single colony

201x1= 201 pooled culture

20 randomly selected patients

1 randomly single colony culture from each patient for WGS
